# Decoding the Equine Genome: Lessons from ENCODE

**DOI:** 10.3390/genes12111707

**Published:** 2021-10-27

**Authors:** Sichong Peng, Jessica L. Petersen, Rebecca R. Bellone, Ted Kalbfleisch, N. B. Kingsley, Alexa M. Barber, Eleonora Cappelletti, Elena Giulotto, Carrie J. Finno

**Affiliations:** 1 Department of Population Health and Reproduction, School of Veterinary Medicine, University of California-Davis, Davis, CA 95616, USA; scpeng@ucdavis.edu (S.P.); rbellone@ucdavis.edu (R.R.B.); nbkingsley@ucdavis.edu (N.B.K.); 2 Department of Animal Science, University of Nebraska, Lincoln, NE 68583-0908, USA; jessica.petersen@unl.edu (J.L.P.); alexa.barber@huskers.unl.edu (A.M.B.); 3 Veterinary Genetics Laboratory, School of Veterinary Medicine, University of California, Davis, CA 95616, USA; 4 Department of Veterinary Science, Gluck Equine Research Center, University of Kentucky, Lexington, KY 40503, USA; ted.kalbfleisch@uky.edu; 5 Department of Biology and Biotechnology “L. Spallanzani”, University of Pavia, 27100 Pavia, Italy; eleonora.cappelletti@unipv.it (E.C.); elena.giulotto@unipv.it (E.G.)

**Keywords:** FAANG, gene regulation, horse, functional annotation, transcriptome, epigenetics, welfare, health

## Abstract

The horse reference genome assemblies, EquCab2.0 and EquCab3.0, have enabled great advancements in the equine genomics field, from tools to novel discoveries. However, significant gaps of knowledge regarding genome function remain, hindering the study of complex traits in horses. In an effort to address these gaps and with inspiration from the Encyclopedia of DNA Elements (ENCODE) project, the equine Functional Annotation of Animal Genome (FAANG) initiative was proposed to bridge the gap between genome and gene expression, providing further insights into functional regulation within the horse genome. Three years after launching the initiative, the equine FAANG group has generated data from more than 400 experiments using over 50 tissues, targeting a variety of regulatory features of the equine genome. In this review, we examine how valuable lessons learned from the ENCODE project informed our decisions in the equine FAANG project. We report the current state of the equine FAANG project and discuss how FAANG can serve as a template for future expansion of functional annotation in the equine genome and be used as a reference for studies of complex traits in horse. A well-annotated reference functional atlas will also help advance equine genetics in the pan-genome and precision medicine era.

## 1. The Horse Genome

The horse reference genomes (Equcab2.0 [1] and EquCab3.0 [2]) are based on a Thoroughbred mare *Twilight* and remain the only high-quality genome assemblies for equids. EquCab2.0 has 42,304 gaps comprising 55 Mb (2.2% of the genome) in total, with a scaffold N50 of 46 Mb. In comparison, EquCab3.0 contains 3771 gaps comprising 9 Mb (0.34% of the genome) with a scaffold N50 of 86 Mb. It has 99.7% mammalian Benchmarking Universal Single-Copy Orthologs (BUSCO) (5 fragmented and 7 missing out of 4104 mammalian universal orthologs), compared to that of 99.0% (4064 complete orthologs) in EquCab2.0 [2]. Owing to the availability of a high-quality reference genome sequence, researchers have been able to utilize a wide variety of high-throughput tools to interrogate genetic etiologies for various equine traits. Recently, Raudsepp et al. provided a comprehensive review of major discoveries using combinations of recent technologies including genome-wide association studies (GWAS), whole-genome sequencing (WGS), and RNA-seq [3]. 

Using these tools, successful identification of the genetic variants responsible for simple Mendelian traits have been identified, including a novel variant in glutamate metabotropic receptor 6 (*GRM6*) associated with congenital stationary night blindness [4] and a nonsense variant in rap guanine nucleotide exchange factor 5 (*RAPGEF5*) associated with equine familial isolated hypoparathyroidism [5]. However, many GWA studies conducted in horses have identified significant regions of association that do not contain any known genes. In humans, it was estimated that 88% of trait/disease associated single nucleotide polymorphisms (SNPs) identified from GWAS were either intergenic or intronic [6]. These SNPs would later be recognized as enriched in various functional elements [7]. Since then, numerous studies have examined different mechanisms by which noncoding variants may affect phenotype. Variants near these significantly associated SNPs have been found to create transcription factor (TF) binding sites [8], disrupt binding motifs [9], or alter TF binding affinities [10,11].

These findings support the notion that many noncoding regions of DNA have important regulatory functions that affect gene expression. With a comprehensive registry of 926,535 human regulatory elements [12], it is now common to include functional annotation in the fine mapping of traits post-GWAS [13]. However, no such resources are available for most animal species, including horses. To address this critical gap in knowledge, FAANG was proposed as an effort to identify important regulatory elements in the major livestock species [14].

## 2. Functional Annotation of Animal Genomes

The ENCODE initiative was proposed in 2003 as an ambitious effort to “identify all functional elements in the human genome sequence” [15]. In 2017, ENCODE concluded its third phase, delivering an integrated set of DNA transcription, regulation, and epigenetic modifications from a total of 7495 experiments in more than 500 cell types and tissues [12].

After almost two decades, ENCODE improved our understanding of gene regulation and delivered a wide range of computational tools, as well as a rich deposit of well-documented, publicly available experimental datasets [12]. Inspired by its phenomenal success, an international group of researchers proposed a similar, coordinated effort to systematically annotate animal genomes, providing vital resources to animal genetics research communities, termed Functional Annotation of Animal Genomes (FAANG) [14]. As part of the FAANG initiative, the equine FAANG group has been actively working with the larger FAANG community and ENCODE researchers to lead the annotation efforts for the horse genome.

The first stage of the equine FAANG initiative was to generate a biobank of reference tissues from comprehensively phenotyped animals. Burns et al. [16] and Donnelly et al. [17] detailed the phenotyping of four selected reference animals (UCD_AH1 – UCD_AH4) and a collection of over 80 tissues from each individual. These healthy animals were selected from the same breed (Thoroughbred) as Twilight, the horse used to construct the equine reference genome. When considering selection for the FAANG horses, the priority was placed on representing healthy Thoroughbred horses. Because *Twilight* was selected for the equine reference sequence based on homozygosity across the equine leukocyte antigen (ELA) region [1], the decision was made to include three unrelated Thoroughbreds and one (AH4) half-sibling of *Twilight* to achieve this goal while still aligning well with the reference sequence. A unique aspect of this biobank is that horses were extensively phenotyped, both antemortem by experienced veterinarians and postmortem by veterinary pathologists. This not only ensured that there was no evidence of clinical or subclinical disease in these animals, but it also provided insight into the cellular composition of the tissues selected for assays. These tissues are stored at −80 °C in a biobank at UC Davis and are available to all equine FAANG researchers.

Here, we briefly discuss some of the most relevant findings from ENCODE and their implications for functionally annotating the equine genome.

## 3. Transcriptome

The transcriptome is the collection of all transcripts in an organism. It includes protein-coding mRNAs as well as noncoding RNAs. During the second phase of ENCODE, 62% of the human genome was found to be transcribed with 31% of transcribed bases located in intergenic regions [18]. Many of these transcripts have been recognized as noncoding RNAs with important regulatory roles [19,20,21,22,23]. Additionally, in any cell line, 39% of the genome was transcribed on average. Up to 56.7% of transcriptome was detected in at least one of fifteen studied cell lines. Interestingly, only 7% of protein-coding genes were cell-line specific, while 53% were constitutive. In comparison, long-noncoding RNAs (lncRNAs) appeared to contribute more to cell-line specificity, with 29% of lncRNAs detected in only one of the fifteen studied cell lines and 10% expressed in all cell lines [18]. These results highlighted the necessity of characterizing transcriptome in a cell-specific manner. 

As part of ENCODE, GENCODE was initially founded to provide high-quality reference gene annotation for the human genome and subsequently expanded into a long-running partnership between several groups and institutes. In its most recent release based on GRCh38, a total of 60,649 genes have been identified in the human genome, of which 19,955 are protein coding, with an average isoform-to-gene ratio of 3.9 [24]. It was also demonstrated that genes tend to express many isoforms simultaneously, with a dominant isoform comprising 30% or more of its corresponding gene expression. Isoforms also appeared to contribute to cell type specificity, with over 75% of protein-coding genes having different dominant isoforms in different cell lines [18]. 

In addition to protein-coding transcripts, the transcriptome also consists of many noncoding RNA species, including both small and long noncoding RNAs. The functions of these RNAs have been extensively examined and implicated in important biological pathways [25,26,27,28]. The small noncoding RNAs present a unique opportunity to new therapeutic approaches [29]. Extensive efforts have been put into cataloguing noncoding RNAs in the human and mouse genome [30,31]. These efforts have further detailed the extent of noncoding RNA regulatory network and the diversity of noncoding RNA species and their functions. 

Taken together, these findings from ENCODE demonstrated the importance of noncoding RNAs and of alternative splicing in cell-specific expression and regulation. Both Ensembl [32] and RefSeq [33] provide noncoding RNA and isoform annotation for EquCab3.0 by utilizing the high-quality annotation of the human genome as well as publicly available horse RNA-seq data. RefSeq annotation for EquCab3.0 consists of 30,022 genes, of which 21,129 are protein coding, with an average isoform-to-gene ratio of 2.6 [34]. The Ensembl annotation of the equine genome contains 30,371 genes (20,955 protein coding) with an average isoform-to-gene ratio of 1.9 [35]. Assuming the human and equine genomes have a similar number of genes and consistent isoform-to-gene ratio, the current horse gene annotation likely lacks many noncoding RNAs and alternate isoforms. 

The FAANG initiative proposed RNA-seq assays for both mRNA and smRNA to identify and quantify these transcripts in a tissue-specific manner [14]. These assays have been performed for eight prioritized tissues (liver, lamina, heart, parietal cortex, adipose, skeletal muscle, ovary/testis, and lung) (Table 1).

To facilitate data generation for the remaining biobanked tissues, we proposed a unique “Adopt-A-Tissue” model for mRNA-seq. Researchers were invited to “adopt” a tissue or tissues fitting their research interests, which meant they would cover the assay and sequencing costs. All library preparations and sequencing were performed at the same two locations (female samples at UC Davis, male samples at University of Nebraska-Lincoln) to minimize variability. This approach allowed the community to contribute to the initiative together while still being able to limit technical variations across laboratories during library constructions [36]. Owing to this unique strategy, the equine community has sequenced over 40 tissues, and the data have been made publicly available (Table 1).

More recently, long-read sequencing assays such as PacBio Isoform sequencing (Iso-seq) have emerged as powerful tools to determine the splicing patterns of transcripts. To address the poor isoform annotations currently available for the horse genome, Iso-seq assays are being performed in 8 tissues (liver, lung, lamina, heart, ovary, testis, muscle, skin, and parietal cortex) across eight PacBio Sequel 8M SMRT cells. By combining a wide variety of assays, the equine FAANG initiative aims to deliver a comprehensively annotated transcriptome for the horse genome.

## 4. Chromatin Accessibility

In mammalian cells, DNA molecules are packed by histone proteins to form nucleosomes and are subsequently compacted into chromatin [37,38]. Compact chromatin restricts access to DNA molecules by transcription factors and serves as a way to regulate gene expression [39]. For example, nucleosomes are densely arranged in facultative and constitutive heterochromatin while depleted in active regions such as active enhancers, insulators, and transcribed gene bodies [40,41]. Using DNase-seq, a DNase I assay quantifying susceptibility of chromatin to DNase I, Boyle et al. identified 94,925 DNase I hypersensitive sites (DHS) covering 2.1% of the human genome [42]. It was also found that only 13% of DHS were located within promoters, while up to 78% were in intergenic or intronic regions. Remarkably, DHS were found in or near the transcription start sites (TSS) of nearly all highly expressed genes. However, while DNase I hypersensitivity appeared to be necessary for gene expression, it was not sufficient as DHS were also observed in unexpressed genes [42]. The association between accessible chromatin and active elements present a unique opportunity to study tissue- and cell-specific gene regulation [43,44,45,46,47]. 

Echoing their strong functional implications, accessible chromatin was also shown to be associated with noncoding variants identified in GWAS studies of common traits. Maurano et al. examined 5654 noncoding variants identified in the GWAS studies of 207 diseases and 447 quantitative traits and found 76.6% of these variants lie either within a DHS or in complete linkage disequilibrium (LD) with another SNP in DHS [48]. The data further demonstrated that many of these DHS were strongly correlated with the promoter of a distal gene target [48]. Gusev et al. analyzed the heritability of 11 common diseases and found that SNPs contained within DHS explained up to 79% of heritability [49]. The strong association between accessible chromatin and functional elements warranted efforts to establish a catalog of tissue-specific DHS to facilitate discoveries of functionally relevant variants [47].

Although DNase-seq has proven successful in identifying accessible chromatin, its laborious protocol, slow turn-around time, and large sample size requirements severely limit large-scale applications [50,51]. Buenrostro et al. developed Assay for Transposase-Accessible Chromatin with high-throughput sequencing (ATAC-seq), which greatly reduced both time and labor costs while requiring lower nuclei input [51]. Owing to its simple protocol and comparable output [52], ATAC-seq has been widely adopted as a state-of-the-art method for interrogating genome-wide chromatin accessibility; further, several variations in methodology have been developed to apply ATAC-seq to frozen tissues [53], cryopreserved nuclei [54], or to improve sensitivity in low-input materials [55]. 

Using ATAC-seq on cryopreserved nuclei from eight tissues across pig, cattle, and mouse, Halstead et al. showed a lack of conservation of sequence and accessibility in accessible sites across evolutionary distance, with 20% shared sites between pig and cattle and only 10% between mouse and ungulates [56]. Therefore, it is necessary to establish a tissue-specific catalog of accessible sites specifically for the horse genome. A pilot study was recently carried out to evaluate the suitability of frozen equine tissue derived nuclei for ATAC-seq [57]. Following protocols established by this study, additional ATAC-seq experiments are underway to expand this assay to eight prioritized tissues for the equine FAANG project.

## 5. Histone Modifications

Histone proteins form the basic building blocks of hierarchical chromatin structures and have been recognized to play an important role in modulating gene expression through post-transcriptional modifications [58,59,60,61]. A nucleosome core is formed by two copies of each of the four major types of histone proteins: H2A, H2B, H3, and H4 [62]. Since Allfrey first suggested the potential role of histone acetylation in regulating gene expression in 1964 [58], extensive research has been carried out to understand the roles, mechanisms, and implications of different histone modifications. Histone 3 lysine 4 monomethylation (H3K4me1), H3K4me3, H3K27me3, and H3K27ac are among some of the most studied and best understood modifications. Hyun et al. provided a detailed review of molecular mechanisms associated with histone lysine modifications and their regulatory functions [63]. Here, we briefly discuss ENCODE findings regarding histone marks and how they can be integrated to provide a more comprehensive view of regulatory activities.

Barski et al. first comprehensively assayed histone modifications across the human genome using high-throughput sequencing [64]. Consistent with previous studies, H3K4 methylation marks were enriched in promoter regions. A significant drop in signal between −200 bp and +50 bp of TSS was observed for H3K4me3 with major peaks at −300 bp and +100 bp [64]. This was consistent with observations that H3K4me3 was primarily associated with promoter regions [65] and that nucleosomes were depleted near active TSS [40]. On the other hand, H3K4me1 showed a distinct bimodal signal with peaks around –900 bp and +1000 bp of TSS [64], in agreement with previous observations that H3K4me1 was enriched in enhancer regions [66]. Similarly, H3K27me3 was observed at a higher level around the TSS of silent genes than those around active genes, supporting correlation between H3K27me3 and gene repression [67]. Conversely, H3K27ac was observed around active elements and associated with higher expression level [68].

Taken together, the four histone modifications discussed in this manuscript represent major regulatory elements and can provide valuable information regarding tissue-specific regulatory activities in the horse genome. Using genome-wide chromatin immunoprecipitation sequencing (ChIP-seq) for these four marks in eight prioritized tissues in the two female FAANG horses, Kingsley et al. reported over one million putative regulatory sites [69]. The utility of these data were demonstrated when a 16 kB intergenic deletion associated with an ocular condition in horses, namely distichiasis, was discovered and FAANG ChIP-seq data showed that this region harbors a tissue specific active enhancer [70]. Undoubtedly, these data will continue to aid in the understanding of other structural variants causing or associated with disease in the horse as additional tissues are evaluated. Following the success of the mRNA Adopt-A-Tissue initiative, similar efforts have facilitated characterization of histone marks in four tissues important to equine health and traits of economic impact (spleen, metacarpal 3, sesamoid, and skin) [71]. Furthermore, additional Adopt-A-Tissue efforts are currently ongoing to facilitate histone ChIP-seq assays for the remaining FAANG tissues.

## 6. CTCF Binding

CCCTC-binding factor (CTCF) is a well-studied zinc finger protein that serves a central role in the formation of chromatin topology and remodeling. It was first discovered as a repressive transcription factor in chicken for c-*MYC* [72] as well as *LYZ* [73]. It was later shown that CTCF may also serve as an activator for the Amyloid β-Protein Precursor gene (*APP)* [74]. In 1999, Bell et al. reported a CTCF binding site at the core of an insulator element at the 5′ end of the chicken β-globin gene *HBB* [75]. Insulators are genomic regions that separate genes from cis-regulatory elements [76]. This site also sits at a boundary between active and inactive chromatin [77], a typical feature of an insulator element [78,79]. 

Many seemingly contradictory functions of CTCF have attracted extensive efforts to understand the mechanisms of its multivalent roles. CTCF is highly conserved across species [80,81] and embryonically lethal when knocked out in mice [82]. The binding motif of CTCF consists of a ~20 bp core consensus sequence and less conserved peripheral sequences, comprising ~50 bp [83,84]. ChIP assays targeting CTCF revealed several unique patterns. First, CTCF binding sites were observed across the genome, with over 40% within intergenic regions [64,83,85]. Consistent with the insulator activity of CTCF, two distinct types of loci with opposing CTCF binding patterns were observed. Loci depleted of CTCF binding sites tend to include clusters of related gene families and transcriptionally coregulated genes, while loci enriched in CTCF binding sites tend to have genes with alternative promoters [83]. Furthermore, CTCF was shown to be crucial for chromatin loop formation at the mouse β-globin locus [86]. Similarly, Hou et al. described an alternative loop formation by inserting a CTCF binding insulator HS5 between the β-globin locus and its upstream locus control region [87]. Additionally, cohesin has been functionally associated with CTCF in mediating chromatin loops [88,89]. These results suggested a potential mechanism via which CTCF mediates regulation of chromatin conformation and gene expression. 

The introduction of Hi-C technology that enabled genome-wide interrogation of long-range interactions [90] quickly brought about new insights into the mechanisms of CTCF function. Refining the resolution of the Hi-C interaction maps to kilobases, Rao et al. observed that the majority of chromatin loops were associated with convergent pairs of CTCF motifs, as well as colocalizing with cohesin proteins [91]. The orientation of CTCF motifs was also shown to determine the directionality of the CTCF mediated interactions [92]. Finally, the significance of such directionality was functionally demonstrated by inverting CTCF sites with CRISPR to alter genome topology as well as promoter function [93]. 

These findings led to a proposed extrusion model [94,95], where a chromatin loop is pulled through an extrusion complex consisting of cohesin and CTCF and is stabilized by a CTCF dimer. This model explains the convergence of a CTCF pair surrounding a chromatin loop, as well as the many regulatory functions of CTCF observed in early studies. More evidence is emerging in support of this model. Based on this model, Fudenberg et al. used simulation to reproduce topologically associated domains (TADs) and contact frequencies observed in Hi-C studies as well as to recapitulate experimental results where TADs were observed to spread upon depletion of CTCF binding sites [96]. Haarhuis et al. showed that cohesin release factor WAPL could restrict chromatin loop extrusion by releasing cohesin from DNA and that knocking out *WAPL* results in enlarged chromatin loops between incorrectly orientated CTCF motifs [97]. Allahyar et al., employing a multi-contact 4C technology, showed that such enlarged loops in *WAPL* knockout cells are a result of aggregated CTCF loop anchors, or a “cohesin traffic jam” [98].

Given its central role in chromatin loop formation, CTCF binding sites can be considered an intermediate between the 1D genomic sequence and 3D chromatin topology. Although there is no simple rule to determine the functional outcome of a disrupted CTCF binding site, as it largely depends on its interaction with surrounding regulatory elements, there is no doubt that a catalog of CTCF binding sites in a given cellular context can provide valuable information when decoding the functional implications of DNA variants. 

Following the practices established by the FAANG community, characterization of CTCF binding sites using ChIP-seq is being performed on eight prioritized tissues for both sexes. Analyses to identify both tissue and sex-specific CTCF binding and integrate all of the FAANG ChIP-seq data into chromatin state annotations are currently underway.

## 7. Chromatin States

While the associations between individual histone marks and regulatory activities are noteworthy, combinations of histone marks have proven to be more reliable in the fine-scale predictions of regulatory elements. For example, Creyghton et al. observed that the H3K27ac mark could distinguish active enhancers from inactive/poised enhancers, which are both marked by H3K4me1 [68]. Bernstein et al. similarly identified a bivalent signal with both H3K4 methylation and H3K27 methylation, suggesting a poised regulatory element [99]. These findings prompted hypotheses that various regulatory functions of noncoding DNA could be explained by either additive properties [100] or unique combinations of histone modifications [101]. New unsupervised computational approaches were subsequently developed to classify histone modification patterns and partition them into different chromatin states [102,103]. Ernst et al. identified 11 promoter states, all marked by H3K4me3 and varying presence and levels of several other marks, as well as 4 enhancer-associated states, all marked by H3K4me1 and varying frequencies of acetylation marks [103]. These findings suggest that some histone modifications (H3K4me1, H3K4me3) designate unique regulatory elements while other modifications (acetylation marks including H3K27ac) enhance regulatory activity in an additive fashion. 

The recognition of chromatin states and introduction of computation tools such as ChromHMM [104] provided a way to systematically profile the regulatory landscape in any given cellular context. Taking advantage of this development and the availability of ChIP-seq data from the four major histone marks and CTCF, efforts to compose an integrated tissue-specific chromatin state map are currently underway for the equine genome.

## 8. Unique Aspects of the Horse Genome

Centromeres are enigmatic structures because, contrary to other genetic loci, their function is not determined by the underlying DNA sequence but depends on epigenetic factors. The Centromere Protein A (CENP-A) is a centromere-specific variant of histone H3 that epigenetically identifies, maintains, and propagates centromere function [105]. The characteristics of its binding domain have been elusive to investigators due to its typical association with tandemly repeated DNA (satellite DNA). In this context, a turning point was the discovery that the centromere of horse chromosome 11 (ECA11) was completely devoid of satellite DNA, demonstrating for the first time that a natural mammalian centromere, fixed in a species, can exist without satellite sequences [1]. Owing to the lack of satellite repeats at the centromere of ECA11 and the availability of the horse reference genome, the genomic position of the corresponding CENP-A binding domain could be precisely identified by ChIP-on-chip with an anti-CENP-A antibody [1]. Later, several satellite-less centromeres were identified by ChIP-seq in the donkey genome [106]. These peculiar centromeres found in equid species represent an immature stage of “centromerization”, being the result of centromere repositioning, which is the movement of the centromeric function without detectable chromosomal rearrangements. This event was exceptionally frequent during the rapid evolution of the genus *Equus* [107,108,109]. Such centromeres, being uncoupled from satellite DNA, provide a unique model for dissecting the molecular structure of the centromere [110]. 

The position of the ECA11 satellite-less centromere, identified as the CENP-A binding domain, is not fixed in the horse population but slides within an about the 500 kb region, giving rise to different positional alleles or “epialleles” [106,111,112]. The analysis of these epialleles carried out on families composed by horses, donkeys, and their hybrid offspring (mule/hinny) revealed that they are inherited as Mendelian traits, but their position can slide in one generation [106]. Conversely, the position of the centromere is stable during mitotic propagation of cultured cells grown for several population doublings, suggesting that the sliding may presumably take place during meiosis or early embryogenesis [106].

The absence of satellite DNA at these centromeres also provides a unique opportunity to understand whether some typical features of mammalian centromeres depend on the presence of satellite DNA. In particular, it was possible to demonstrate that satellite DNA was not necessary for segregation fidelity of the centromere [113] and was not implicated in the suppression of meiotic recombination, which is typically exerted by the centromere [112].

The rich repository of tissues from different developmental origins available through the FAANG project will allow us to answer other important questions on centromere biology using the ECA11 centromere as model system. We will test whether the centromere position is conserved during development or if it can slide during tissue differentiation. In addition, thanks to the large amount of data regarding the functional annotation of the horse genome, generated within the FAANG effort, we will be able to map the epigenetic marks available through the consortium in the ECA11 centromeric region. The results will indicate whether chromatin markers and transcriptional activity at ECA11 centromere vary across tissues and individuals, and with respect to centromere position. Furthermore, CENP-A has been shown to bind at TF binding sites and promoters, suggesting potential regulatory activities [114]. Therefore, utilizing FAANG data, we will be able to identify the regulatory activities of CENP-A and any roles centromeres may play during tissue differentiation

## 9. Summary and Future Perspectives

Just three years after starting the tissue and data collection for the equine FAANG initiative, the community has completed over 400 experiments from more than 50 tissues using a variety of assays targeting different features of the horse regulatory landscape (Table 2). Data are being made available to the public as they are generated and evaluated for passing quality control measures; these data have been and continue to be utilized in unrelated research projects [5,70,115]. Integrated analysis is currently ongoing to provide a systematic annotation of major functional elements in the horse genome available, as a central hub hosted on UCSC genome browser to the research community. 

With over 80 tissues collected from four healthy and comprehensively phenotyped animals, we will be able to generate a map of gene expression and regulation throughout the horse body, providing unique opportunities to investigate tissue-specific gene expression and gene networks. However, this tissue collection presents a serious challenge for data analyses. Heterogeneity both within tissues as a result of cell-type differentiation and across tissues as a result of tissue infiltration or contamination during collection, can confound analysis of tissue-specific expression and regulation. The prevalence of this issue was recently reported by Sturm et al. [116]. To mitigate this issue, careful histological assessment was performed during the tissue collection phase to minimize the possibility of tissue infiltration or contamination. However, caution should be taken to assess the extent of tissue heterogeneity during data analysis. Additionally, single-cell based technologies have proven useful to profile cell types from complex tissues [117,118,119,120], and the adoption of these technologies to equine FAANG data are being discussed within the community and will likely be integrated in the next steps of the multi-phased approach of this project.

While the equine FAANG biobank represents a wide variety of tissue types, the four horses these tissues were collected from represent only a narrow subset of the horse population, as well as developmental stages. These horses were intentionally selected to be of the same breed as the reference genome assembly in order to better annotate the reference genome assembly. However, caution should be taken with interpretation and extrapolation of these data to other breeds or developmental stages. Regardless, this initiative will serve as a template and reference point for the future expansion of the transcriptome and epigenome of equids. 

FAANG represents a notable international collaborative effort in the equine community that has brought together equine researchers and practitioners from around the globe. Most importantly, FAANG collaborators have been vocal proponents of open science and broad data accessibility within the equine community. The growing number of publicly available datasets is accelerating discoveries and powering large-scale analyses. Well-annotated and carefully documented FAANG data with accompanying comprehensive metadata will serve as a reference point for many future discoveries in horse.

## Figures and Tables

**Table 1:** Overview of Available Data and Assay Details.

Project Accession	Assay	Samples	Tissues	Instrument	Library Layout	Number of Experiments
PRJEB26698	WGS	Two females	1	HiSeq 2500 (San Diego, CA, USA)	2 × 250 bp	2
PRJEB42407	WGS	Two males	1	NovaSeq 6000 (San Diego, CA, USA)	2 × 150 bp	2
PRJEB26787	RNA-seq	Two females	30	HiSeq 2500 (San Diego, CA, USA)	2 × 250 bp	60
PRJEB32645	RRBS	Two females	10	HiScanSQ (San Diego, CA, USA)	1 × 50 bp	20
PRJEB35307	Histone ChIP-seq	Two females	8	HiSeq 4000 (San Diego, CA, USA)	1 × 50 bp	80
PRJEB42315	Histone ChIP-seq	Two females	4	HiSeq 4000 (San Diego, CA, USA)	1 × 50 bp	38
PRJEB41079	CTCF ChIP-seq	Two females	8	HiSeq 4000 (San Diego, CA, USA)	1 × 50 bp	28
PRJEB41317	ATAC-seq pilot	Two females	2	HiSeq 4000/NextSeq 500 (San Diego, CA, USA)	2 × 75 bp/2 × 42 bp	16

WGS: whole-genome sequencing; RNA-seq: mRNA sequencing; RRBS: reduced-representation bisulfite sequencing; Histone ChIP-seq: chromatin immunoprecipitation using sequencing for the four major histone marks; CTCF ChIP-seq: chromatin immunoprecipitation using sequencing for CTCF protein; ATAC-seq pilot: assay for transposase accessibility using sequencing.

**Table 2:** Overview of Completed Assays.

Assay	Animals	Tissue Types	Total Experiments
WGS	AH1-AH4	Blood	4
mRNA-seq	AH1	47	140
	AH2	46	
	AH3	23	
	AH4	24	
Iso-seq	AH1–AH4	12	48
ChIP-seq–H3K4me1	AH1–AH2	12	40
	AH3–AH4	8	
ChIP-seq–H3K4me3	AH1–AH2	12	40
	AH3–AH4	8	
ChIP-seq–H3K27ac	AH1–AH2	12	40
	AH3–AH4	8	
ChIP-seq–H3K27me3	AH1–AH2	12	40
	AH3–AH4	8	
ChIP-seq–CTCF	AH1–AH2	8	32
	AH3–AH4	8	
ATAC-seq	AH1–AH4	10	40
RRBS	AH1–AH2	10	20
smRNA-seq	AH1–AH2	48	96
Total		48	444

## Data Availability

All FAANG data discussed in this manuscript can be accessed from Sequence Read Archive (SRA), European Nucleotide Archive (ENA), or faang.org/dataset using accession numbers listed in Table 1.
